# Polarization-Multiplexed High-Throughput AOTF-Based Spectral Imaging System

**DOI:** 10.3390/ma16124243

**Published:** 2023-06-08

**Authors:** Hao Zhang, Huijie Zhao, Qi Guo, Dong Xu, Wenjie Teng

**Affiliations:** 1 School of Instrumentation Science and Opto-Electronics Engineering, Beihang University, Beijing 100191, China; hao2017@buaa.edu.cn (H.Z.);; 2 Aerospace Optical-Microwave Integrated Precision Intelligent Sensing, Key Laboratory of Ministry of Industry and Information Technology, Beihang University, Beijing 100191, China; 3 Institute of Artificial Intelligence, Beihang University, Beijing 100191, China

**Keywords:** AOTF, spectral imaging system, polarization multiplexed, high throughput

## Abstract

Spectral imaging detection using acousto-optical tunable filters (AOTFs) faces a significant challenge of low throughput due to the traditional design that only receives a single polarization light. To overcome this issue, we propose a novel polarization multiplexing design and eliminate the need for crossed polarizers in the system. Our design allows for simultaneous collection of ±1 order light from the AOTF device, resulting in a more than two-fold increase in system throughput. Our analysis and experimental results validate the effectiveness of our design in improving system throughput and enhancing the imaging signal-to-noise ratio (SNR) by approximately 8 dB. In addition, AOTF devices used in polarization multiplexing applications require optimized crystal geometry parameter design that does not follow the parallel tangent principle. This paper proposes an optimization strategy for arbitrary AOTF devices which can achieve similar spectral effects. The implications of this work are significant for target detection applications.

## 1. Introduction

**Figure 1:**
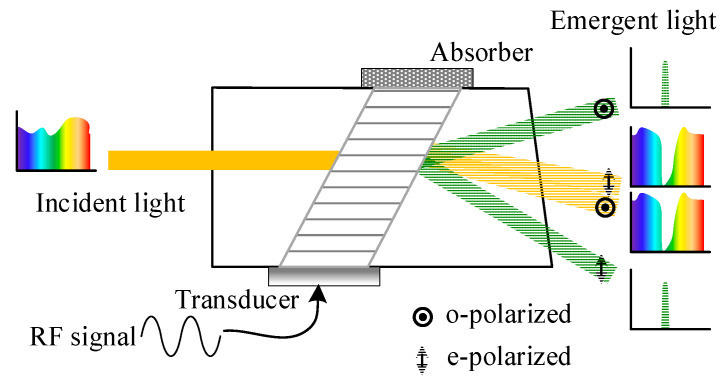
Principle diagram of the AOTF device.

The acousto-optic tunable filter (AOTF) is a narrow-band spectral filter that can be electrically tuned. It operates based on the principle of acousto-optic interaction, where the refractive index of the acousto-optic medium undergoes periodic changes in response to an input acoustic wave [1]. This phenomenon resembles the behavior of a diffraction grating [2]. Figure 1 illustrates the AOTF devices, featuring an acoustic wave generated by the transducer and absorbed by an absorber. By switching the radio frequency (RF) signals applied to the transducer, the AOTF device can scan the spectral regions of interest [3]. The AOTF device offers fast response time, high spectral resolution, and wavelength configurability, making it an ideal choice for various applications, including remote sensing and medical imaging [4,5,6,7,8]. However, the low throughput limits the performance of the AOTF spectral imaging system [9,10]. This limitation stems from the optical aperture constraints of the AOTF devices and the extinction design of the system. The typical optical aperture limitations for the visible AOTF devices with TeO_2_ crystal are a through-aperture of up to 25 × 25 mm^2^ [11] and a real aperture angle of 9° [12]. These constraints are primarily influenced by the properties of the crystal material, and ongoing research explores alternative materials [13,14]. Meanwhile, the extinction design of the system is mainly attributed to the traditional optical design, which only receives a single polarization of light using crossed polarizers. This design choice leads to a significant throughput loss of over 50% [15].

To address the low-throughput issue of AOTF devices, two solutions are commonly employed: non-polarization design and ultrasonic frequency modulation [16,17]. Non-polarization design aims to enhance throughput by eliminating the polarizer from the system. This approach offers distinct advantages over the ultrasonic frequency modulation method, including the avoidance of multi-frequency crosstalk and limitations on acoustic power, as well as the elimination of specific polarization requirements for incident light. In 1999, Voloshinov introduced a design in which the incident light with different polarization states enters the AOTF device at specific angles to ensure that the ±1 order light is imaged at the same position on the detector. This design utilized a monochromatic laser with beam expansion and the incident light passed through a polarization beam splitter (PBS), separating the ordinary (o) light from the extraordinary (e) light. The o light was reflected into the AOTF device at a predetermined angle [18]. However, this design exhibited optical inconsistency and was only suitable for near-axis fields of view. Subsequently, Fang et al. proposed a dual-arm design, with simultaneous collection of ±1 order light using two mirrors behind the AOTF device [19]. For two-dimensional (2D) imaging of the sample, the design was combined with a motorized scanning stage [20]. Nonetheless, this design resulted in low-resolution images or was limited to single-point detection applications. Subsequently, they developed a design that can be used for spectral imaging applications, involving the modulation of the polarization state of both beams entering the AOTF device through the use of a half-wave plate [21]. However, this design significantly compresses the real aperture angle of the AOTF device, which relies on the principle of separating diffracted (±1 order) and transmitted (0 order) light [2]. The suppression of panchromatic transmitted light in spectral imaging systems is crucial, as its intensity is generally much higher than the effective spectral intensity. Additionally, stray light resulting from the phase-delay variation across the application spectrum range of the half-wave plate also affects image quality. Moreover, the front design of the system is based on collimation optics, which does not facilitate the simultaneous detection of ±1 order light (see Section 2.3 for a detailed analysis). Furthermore, Beliaeva et al. analyzed the feasibility of highly efficient tunable light source using a similar principle [22]. In conclusion, the previous AOTF system designs exhibit limitations in spectral imaging applications, including inconsistent spectral response, compressed real aperture angle, transmission stray light interference, and complex structure.

In this paper, we present a high-throughput AOTF system with a polarization multiplexing design for spectral imaging applications. The front telecentric confocal optics with the optimized AOTF device in the system demonstrates its unique suitability for polarization multiplexing applications. To achieve stray light suppression in spectral imaging systems, the rear blocking optics effectively suppress transmitted light while allowing diffracted light to pass unaffected. Compared to the traditional single polarization detection systems, our proposed design enables simultaneous collection of the ±1 order light of the AOTF device and eliminates the need for crossed polarizers, resulting in an over two-fold throughput improvement. Furthermore, the AOTF devices used in polarization multiplexing applications need optimization of crystal geometry parameters, which does not follow the parallel tangent principle. To address this issue, we propose an optimization strategy for arbitrary AOTF devices that can achieve similar goals, avoiding the cost and long manufacturing periods associated with traditional methods. The proposed design and optimization strategy are introduced and discussed in detail in Section 2, with experimental results from desktop systems provided in Section 3 to verify their effectiveness in achieving high throughput.

## 2. Methods and Analysis

### 2.1. Basic Principles of the Noncollinear AOTF Device

**Figure 2:**
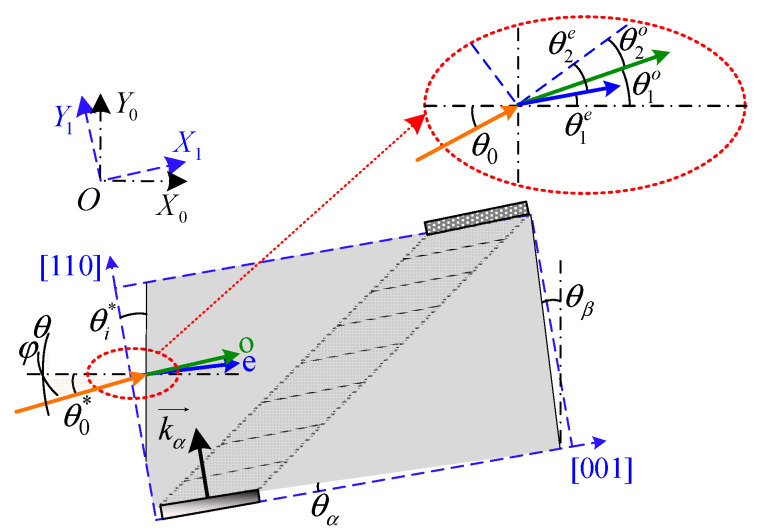
Structure diagram of the noncollinear AOTF device (top view).

There are two fundamental configurations of AOTFs: collinear and noncollinear. In the collinear configuration, the interacting optical and acoustic waves propagate in the same direction, whereas in the noncollinear configuration, the directions of the optical and acoustic waves are different [23]. As shown in Figure 2, a typical noncollinear AOTF device, which usually has three crystal geometry parameters, including the front facet angle ($\theta_{i}^{*}$), the acoustic cut-angle ($\theta_{\alpha }$), and the back facet angle ($\theta_{\beta }$), which greatly affect its performance. In particular, most noncollinear AOTF devices are designed based on the parallel tangent principle to achieve high diffraction efficiency within the angular aperture [24]. For arbitrarily polarized incidence, the o and e light must be analyzed separately due to their distinct refractive indices. In addition, this paper establishes two coordinate systems for analysis: the optical axis coordinate system ($X_{0}OY_{0}$) and the crystal axis coordinate system ($X_{1}OY_{1}$).

### 2.2. Structure of the AOTF Spectral Imaging System

**Figure 3:**
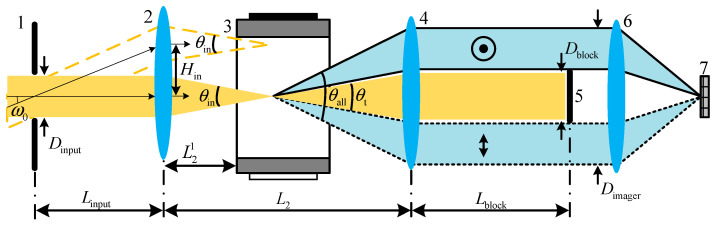
Structure diagram of the proposed AOTF spectral imaging system. 1—input aperture, 2—lens1, 3—AOTF device, 4—lens2, 5—light barrier, 6—lens3, and 7—detector.

**Figure 4:**
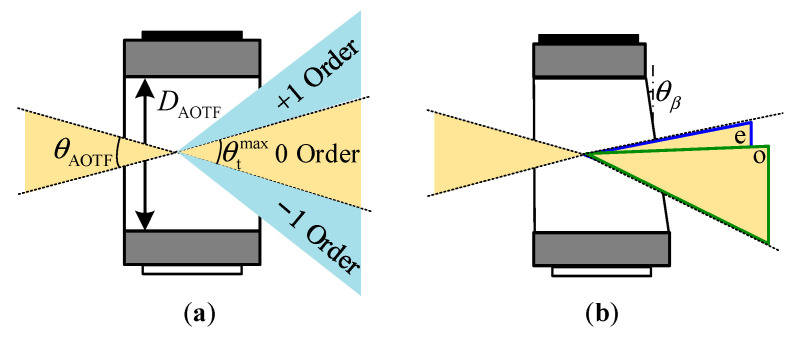
Diagram of AOTF devices. (**a**) AOTF device without a wedge angle for monochromatic light application, and (**b**) the extension of 0 order light caused by the wedge angle in the AOTF device.

The proposed system consists mainly of the front optics, an AOTF device, the blocking optics, and an imager as shown in Figure 3. The AOTF device, which is the central component of the system, has certain constraints on the system design such as the working spectral range, the through aperture ($D_{\mathrm{AOTF}}$), and the real aperture angle ($\theta_{\mathrm{AOTF}}$), as shown in Figure 4a. These constraints affect the design of the front optics. Typically, the front optics have two structures: collimation and confocal [25]. However, in our proposed design, we use telecentric confocal optics, where $L_{\mathrm{input}}$ is equivalent to the focal length ($f_{1}$) of the front lens1 [26,27]. For a $2\omega_{0}$ field-of-view application, the front optics must meet certain requirements as below:

(1) 2f1tanω0=2Hin≤DAOTF2arctanDinput2f1=θin≤θAOTF

In spectral imaging systems, the 0 order light represents the panchromatic spectrum and typically exhibits significantly higher intensity than the effective spectral intensity. Therefore, it is crucial to employ blocking optics to suppress the 0 order light. In this study, the blocking optics are composed of a lens set and a light barrier located at the exit pupil. The width of the light barrier is meticulously designed to fulfill specific criteria:

(2) 2f2tanθtmax2≥Dblock≥2f2tanθt2

where $\theta_{t}$ is the cone angle of the 0 order (panchromatic transmitted) light and $f_{2}$ is the focal length of lens2. Additionally, $\theta_{t}^{\max}$ represents the maximum cone angle of 0 order light, corresponding to the angle between the ±1 order light. This design effectively blocks the 0 order light, while allowing the ±1 order light to pass unaffected. The real aperture angle can be estimated by the separation angle of the normal incident light. It is worth noting that some AOTF devices have a wedge compensation that extends the 0 order light, as depicted in Figure 4b, which should be considered when calculating the real aperture angle.

**Figure 5:**
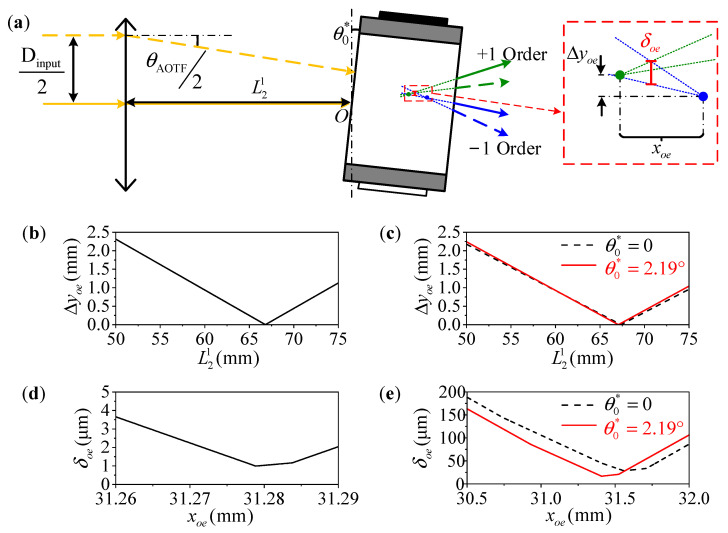
The parameter $L_{2}^{1}$ will affect the relative position of the first image points of the o and e light. (**a**) Schematic diagram of light path. Additionally, the relative position difference distribution is shown with the crystal geometry parameters of (**b**,**d**) $\theta_{i}^{*}=14.14{}^{\circ}$, $\theta_{\alpha }=6.50{}^{\circ}$, and $\theta_{\beta }=0{}^{\circ}$, (**c**,**e**) $\theta_{i}^{*}=15.07{}^{\circ}$, $\theta_{\alpha }=6.49{}^{\circ}$, and $\theta_{\beta }=-4.64{}^{\circ}$. The red solid lines are the results of the 2nd AOTF device by the optimization strategy. $x_{oe}$ in (**d**,**e**) is the relative distance to the incident point O.

The imager is used to capture spectral images and consists of an objective lens and a focal plane array detector. To ensure efficient light collection, the objective lens requires a large entrance pupil diameter, represented as:

(3) Dimager≥2f2tanθall2

where $\theta_{\mathrm{all}}$ is the cone angle of all emitted light as Figure 3. Our proposed system satisfies the “4f” design criteria, where $L_{\mathrm{input}}=f_{1}$, $L_{2}=f_{1}+f_{2}$, and $L_{\mathrm{block}}=f_{2}$. During operation, the incident light forms the first image point inside the AOTF device, and all rear optics perform secondary imaging on this point. We find that the relative position parameter ($L_{2}^{1}$) of the AOTF device is a key parameter, and optimizing this parameter ensures a better image quality of the ±1 order light with polarization multiplexing design. As shown in Figure 5a, the image quality is described by the half-size of the dispersed spot ($\delta_{oe}$) in the diffraction direction of the AOTF device in this paper. The position of the first image point of the o and e light is obtained by ray-tracing the main light and the edge light based on geometric optics theory as Figure 5a, and the ray tracing process inside an AOTF device can be found in Ref. [28]. Then, the traversing method is used to find the optimized $L_{2}^{1}$ so that the lateral difference ($\Delta y_{oe}$) between the two image points is 0 as Figure 5b,c. Finally, the optimal first image point is searched in the interval of o and e light image points to minimize the comprehensive $\delta_{oe}$ of the two beams.

In this paper, two AOTF devices are presented for comparison. The first device (the 1st AOTF) has optimized crystal geometry parameters of $\theta_{i}^{*}=14.14{}^{\circ}$, $\theta_{\alpha }=6.50{}^{\circ}$, and $\theta_{\beta }=0$ (the 1st AOTF) for non-polarization applications [29,30]. The second device (the 2nd AOTF) is used in the experiments and closes to the parallel tangent principle for e-light input application, with crystal geometry parameters of $\theta_{i}^{*}=15.07{}^{\circ}$, $\theta_{\alpha }=6.49{}^{\circ}$, and $\theta_{\beta }=-4.64{}^{\circ}$ [31]. As shown in Figure 5b,d, the optimized $L_{2}^{1}$ of the 1st AOTF device is 66.81 mm, and the minimum $\delta_{oe}$ is less than 1.0 μm. From Figure 5c,e, we can find that the optimized $L_{2}^{1}$ of the 2nd AOTF device is 67.36 mm, and the minimum $\delta_{oe}$ is 28.4 μm. As the optimization strategy is applied to the 2nd AOTF device, resulting in improved aberration values of $L_{2}^{1}=67.06\;\mathrm{mm}$ and $\delta_{oe}=16.7\;\mu m$. Additionally, the optimization strategy is described in detail in Section 2.3. It is observed that using AOTF devices with unoptimized crystal geometry parameters results in large aberrations, which is not desirable for polarization multiplexing design. Additionally, the aberration will be improved with applying the optimization strategy. However, it is pointed out that the optimized AOTF device is a better choice and we are working on this for the future.

### 2.3. Optimization Strategy for Polarization Multiplexing Applications

**Figure 6:**
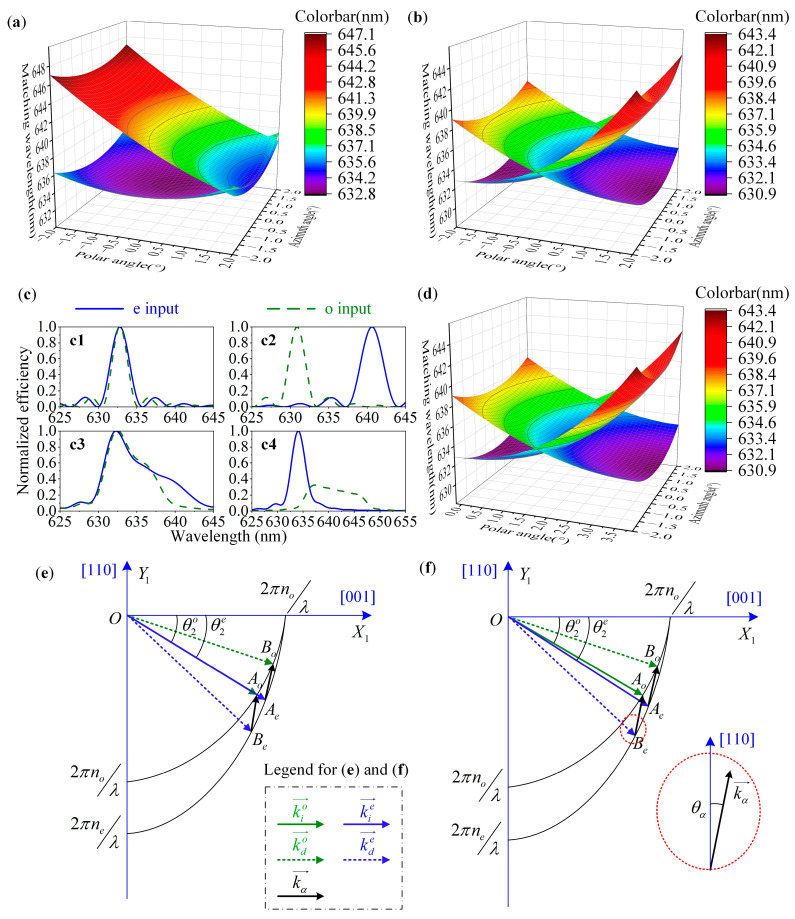
Matched wavelength distributions within the real aperture angle at 632.8 nm of (**a**) the 2nd AOTF device, (**b**) the 1st AOTF device, and (**d**) the 2nd AOTF device by the optimization strategy. (**c**) Spectral curves with the collimation design in which (**c1**) describes the normal incidence of the 1st AOTF device, and (**c2**) corresponds to the edge field. Spectral curves with telecentric confocal design that (**c3**) describes the integral response of the 1st AOTF device, and (**c4**) corresponds to the 2nd AOTF device. (**e**) The wave vector diagram of the 1st AOTF device, and (**f**) corresponds to the 2nd AOTF device by the optimization strategy.

Traditional AOTF devices, which follow the parallel tangent principle, are typically designed for a single polarization state (o or e light). However, when the 2nd AOTF device is directly used for polarization multiplexing applications, there can be a substantial spectral difference in the ±1 order light, as depicted in Figure 6(a,c4). This issue can be mitigated by optimizing the crystal geometric parameters of the AOTF devices [29,30]. By optimizing these parameters, the normal incident o and e light of the same wavelength can satisfy the momentum matching principle at the same tuning frequency, resulting in a significant improvement in the matched wavelength distribution of o and e light within the real aperture angle, as demonstrated in Figure 6b,e. However, developing such a new AOTF device can be costly and time-consuming. Alternatively, we propose an optimization strategy to rotate the traditional AOTF device at an off-axis angle ($\theta_{0}^{*}$) in the polar plane to achieve the same goal, as illustrated in Figure 2. This also enables the o and e light of the same wavelength to satisfy the momentum matching principle at the same tuning frequency, as depicted in Figure 6f. In this case, the matched wavelength distribution of o and e light within the real aperture angle is shown in Figure 6d. The difference between the optimization strategy and the optimized crystal geometric parameters is negligible, with differences of less than 0.01 nm. Moreover, AOTF devices are sensitive to the polarization state and incident angle of the light. It can be observed from Figure 6b,d that the optimized AOTF device can only ensure consistent matched wavelengths of o and e light at the given incident angle. As shown in Figure 6(c1), the optimized AOTF device has the same matched wavelengths of o and e light under normal incidence. Although the matched wavelengths at the edge have been improved, the difference is still about 9.7 nm, as shown in Figure 6(c2). Previous studies have reported the beneficial effects of the telecentric confocal design in suppressing optical sidelobe effects [27]. However, we have found that this design also has a great adaptability for polarization multiplexing applications. It enables the AOTF device to filter the arbitrary views equally, and the spectral response function is equal to the integral within the cone angle of the light entering the AOTF device as [32]:

(4) $$Response\propto \int \int_{-\frac{\theta_{\mathrm{in}}}{2}}^{\frac{\theta_{\mathrm{in}}}{2}}T_{0}^{o}(\theta ,\varphi ,\lambda )I(\eta_{o},L_{o},\Delta k_{o})d\theta d\varphi +\int \int_{-\frac{\theta_{\mathrm{in}}}{2}}^{\frac{\theta_{\mathrm{in}}}{2}}T_{0}^{e}(\theta ,\varphi ,\lambda )I(\eta_{e},L_{e},\Delta k_{e})d\theta d\varphi$$

where θ is polar angle of the incident light and φ is the azimuth angle as shown in Figure 2. $T_{0}^{o}(\theta ,\varphi ,\lambda )$ and $T_{0}^{e}(\theta ,\varphi ,\lambda )$ are the intensities of o and e light entering the AOTF device, respectively. Additionally, I(η,L,Δk) is the diffraction efficiency equation, given as:

(5) $$I=\eta \frac{\sin^{2}{\left[\eta +{\left(\Delta kL/2\right)}^{2}\right]}^{1/2}}{\eta +{\left(\Delta kL/2\right)}^{2}}$$

where L is the acousto-optic interaction length that is affected by the length of the transducer. Additionally, the actual transducer length of the 2nd AOTF device used in the experiments is about 10 mm. The parameter Δk describes the momentum mismatch and η represents the peak diffraction efficiency [33]. By employing telecentric confocal optics, the spectral response curves of o and e light are closer to each other as Figure 6(c3). In contrast, adopting the collimation design would cause the occurrence of “double peaks” in the spectral response curves at some image points as shown in Figure 6(c2), which is not conducive to spectral detection.

The torsional strategy is introduced in detail below. The light with an off-axis angle $\theta_{0}$ obeys the Snell’s law as:

(6) $$n_{0}\sin\theta_{0}=n_{1}\sin\theta_{1}$$

where $\theta_{1}$ is the refraction angle in the crystal, and $n_{0}$ and $n_{1}$ are the refractive indices in the air and crystal, respectively. The $n_{0}$ is generally 1.0 in the air, and $n_{1}$ of the o and e light can be solved by [34]:

(7) $$\left\{\begin{array}{l} n_{1}^{o}=n_{o}\\ n_{1}^{e}=\frac{n_{o}n_{e}}{\sqrt{n_{o}{}^{2}\sin^{2}\theta_{2}^{e}+n_{e}{}^{2}\cos^{2}\theta_{2}^{e}}} \end{array}\right.$$

with markers *o* and *e* to distinguish *o* and *e* light. $n_{o}$ and $n_{e}$ are the principal refractive indices, respectively. In the crystal coordinate system, the refraction angle $\theta_{2}$ is:

(8) $$\theta_{2}=\theta_{1}-\theta_{i}^{*}$$

Using Equations (6)–(8), we can easily obtain $\theta_{2}^{o}$ by:

(9) $$\theta_{2}^{o}=\arcsin(\frac{\sin\theta_{0}}{n_{o}})-\theta_{i}^{*}$$

while $\theta_{2}^{e}$ can be obtained by solving a quadratic equation:

(10) $$\left(\cos^{2}\theta_{i}^{*}-\frac{\sin^{2}\theta_{0}}{n_{e}{}^{2}}\right)\tan^{2}\theta_{2}^{e}+2\sin\theta_{i}^{*}\cos\theta_{i}^{*}\tan\theta_{2}^{e}+\left(\sin^{2}\theta_{i}^{*}-\frac{\sin^{2}\theta_{0}}{n_{o}{}^{2}}\right)=0$$

Now we can obtain $\theta_{2}^{o}$ and $\theta_{2}^{e}$ in the wave vector diagram (Figure 6f) at the incident angle of $\theta_{0}$, and $n_{1}^{o}$ and $n_{1}^{e}$ can also be obtained from Equation (7). The incident optical wave vectors can be solved by:

(11) $$\left\{\begin{array}{l} \left|\vec{k_{i}^{o}}\right|=\frac{2\pi n_{1}^{o}}{\lambda }\\ \left|\vec{k_{i}^{e}}\right|=\frac{2\pi n_{1}^{e}}{\lambda } \end{array}\right.$$

and the coordinates of the intersection A are:

(12) $$\left\{\begin{array}{l} A_{o}=\frac{2\pi n_{1}^{o}}{\lambda }(\cos\theta_{2}^{o},\sin\theta_{2}^{o})\\ A_{e}=\frac{2\pi n_{1}^{e}}{\lambda }(\cos\theta_{2}^{e},\sin\theta_{2}^{e}) \end{array}\right.$$

The linear equations of $A_{o}B_{e}$ and $A_{e}B_{o}$ are similar:

(13) $$y-y_{A}=\frac{1}{\tan\theta_{\alpha }}\left(x-x_{A}\right)$$

The wave vector elliptic equations of o and e light are:

(14) $$\left\{\begin{array}{l} \frac{x^{2}}{n_{o}{}^{2}}+\frac{y^{2}}{n_{o}{}^{2}}={\left(\frac{2\pi }{\lambda }\right)}^{2}\\ \frac{x^{2}}{n_{o}{}^{2}}+\frac{y^{2}}{n_{e}{}^{2}}={\left(\frac{2\pi }{\lambda }\right)}^{2} \end{array}\right.$$

Then, using Equations (12)–(14), the coordinate of the point $B_{e}(x_{B}^{e},y_{B}^{e})$ can be obtained by solving a quadratic equation:

(15) $$\begin{array}{ll} \left(\frac{\tan^{2}\theta_{\alpha }}{n_{o}{}^{2}}+\frac{1}{n_{e}{}^{2}}\right)y_{B}^{e}{}^{2} & +\frac{4\pi \tan\theta_{\alpha }}{\lambda n_{o}}\left(\cos\theta_{2}^{o}-\sin\theta_{2}^{o}\tan\theta_{\alpha }\right)y_{B}^{e}\\ & +\frac{4\pi^{2}}{\lambda^{2}}\left(\cos^{2}\theta_{2}^{o}+\sin^{2}\theta_{2}^{o}\tan^{2}\theta_{\alpha }-2\sin\theta_{2}^{o}\cos\theta_{2}^{o}\tan\theta_{\alpha }-1\right)=0 \end{array}$$

and

(16) $$x_{B}^{e}=\tan\theta_{\alpha }\left(y_{B}^{e}-y_{A}^{o}\right)+x_{A}^{o}$$

Similarly, we can obtain the coordinates of point $B_{o}(x_{B}^{o},y_{B}^{o})$ by:

(17) $$\begin{array}{l} \left(\tan^{2}\theta_{\alpha }+1\right)y_{B}^{o}{}^{2}+\frac{4\pi n_{1}^{e}\tan\theta_{\alpha }}{\lambda }\left(\cos\theta_{2}^{e}-\sin\theta_{2}^{e}\tan\theta_{\alpha }\right)y_{B}^{o}\\ +\frac{4\pi^{2}}{\lambda^{2}}\left[{\left(n_{1}^{e}\right)}^{2}\cos^{2}\theta_{2}^{e}+{\left(n_{1}^{e}\right)}^{2}\sin^{2}\theta_{2}^{e}\tan^{2}\theta_{\alpha }-2{\left(n_{1}^{e}\right)}^{2}\sin\theta_{2}^{e}\cos\theta_{2}^{e}\tan\theta_{\alpha }-n_{o}{}^{2}\right]=0 \end{array}$$

and

(18) $$x_{B}^{o}=\tan\theta_{\alpha }\left(y_{B}^{o}-y_{A}^{e}\right)+x_{A}^{e}$$

Finally, the fitting torsion angle $\theta_{0}^{*}$ can be obtained by traversing method with $\left|\vec{A_{o}B_{e}}\right|=\left|\vec{A_{e}B_{o}}\right|$. When using a normal AOTF device with e light input design, $\theta_{0}^{*}$ is greater than 0. While for an AOTF device with o light input design, $\theta_{0}^{*}$ is less than 0. In this study, the 2nd AOTF device used had a fitting $\theta_{0}^{*}$ of 2.19°. The optimization strategy employed also allows for the same AOTF device to be used in o light input mode, which can be achieved through the calculation of the Bragg angle of the o light input application under the parallel tangent principle. Specifically, the Bragg angle of the o light input application should be 13.18° at $\theta_{\alpha }=6.49{}^{\circ}$, and the torsion angle required is 4.29°, switching to the o light input mode. We can now set the example system parameters as shown in Table 1.

**Figure 7:**
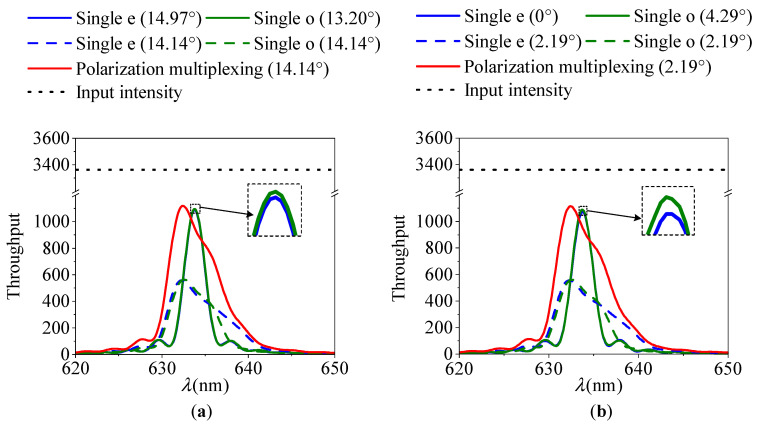
Spectral response curves. (**a**) The 1st AOTF device with optimization of crystal geometric parameters. $\theta_{i}^{*}=14.97{}^{\circ}$ and $\theta_{i}^{*}=13.20{}^{\circ}$ satisfy the parallel tangent principle of e and o light, respectively. (**b**) The 2nd AOTF device with the optimization strategy.

We have further calculated the theoretical throughputs of the two AOTF devices using Equation (4) with the parameters listed in Table 1. The results of these calculations are presented in Figure 7 and Table 2. In our calculations, we accounted for both polarization states and utilized an angular sampling step of 0.1°. As a result, we obtained an input intensity of 3362 (41 × 41 × 2) at each wavelength within the real aperture angle of 4.0°. Compared to the traditional single polarization detection, the polarization multiplexing design proposed in this paper can improve the throughput by about two-fold. Additionally, we observed a slight increase of 0.7% in the peak diffraction efficiency.

## 3. Results and Discussion

### 3.1. Test and Analysis of Optimization Strategy

**Figure 8:**
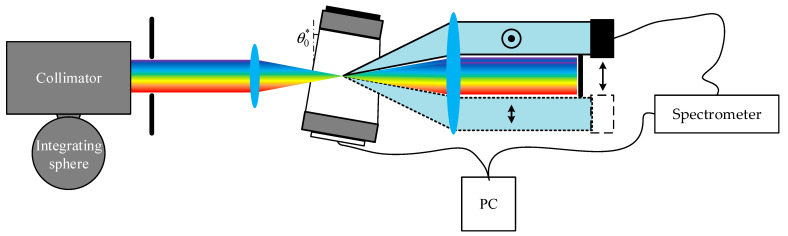
Structure diagram of the desktop system for testing the spectral curves.

**Figure 9:**
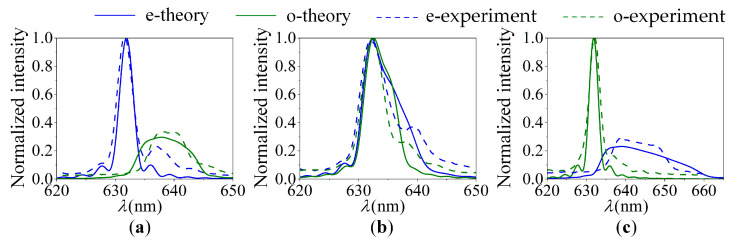
The tested and theoretical spectral response curves at (**a**) $\theta_{0}^{*}=0$, (**b**) $\theta_{0}^{*}=2.19{}^{\circ}$, and (**c**) $\theta_{0}^{*}=4.29{}^{\circ}$.

A spectral test optics setup, as shown in Figure 8, was constructed. The light source was a wide-spectrum light source composed of an integrating sphere (Halogen lamp) and a collimator. The input aperture used is a circular aperture of 5.2 mm diameter. The AOTF device selected for the experiment was the SGL100-400/850-20LG-K, which was sourced from China. The commercial spectrometer used is the Ocean Optics USB2000+. Spectral sampling of o and e light was performed behind the light barrier (exit pupil). The results of spectral tests are shown in Figure 9. Additionally, the results show good agreement with the theoretical calculations. This demonstrates that spectral modulation of the AOTF device can be realized using the optimization strategy, making it suitable for polarization multiplexing applications. In addition, our previous work has revealed the consistent response of the incident light at a torsion angle of 2.19° [31].

### 3.2. Test and Analysis of Throughput

**Figure 10:**
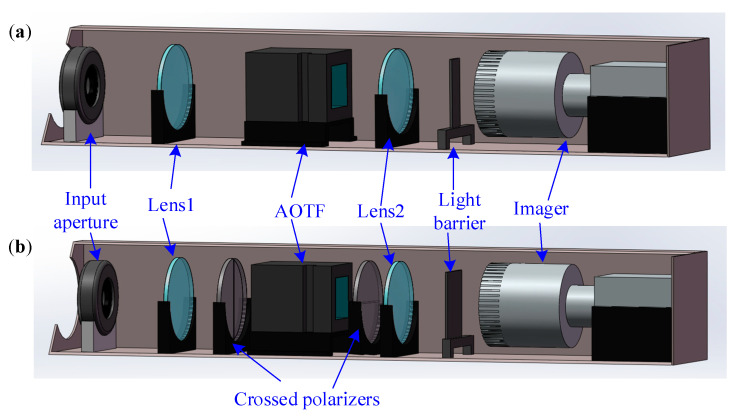
Structure diagram of the AOTF spectral imaging systems for testing the throughput. (**a**) Polarization multiplexing design. (**b**) Single polarization detection design.

**Figure 11:**
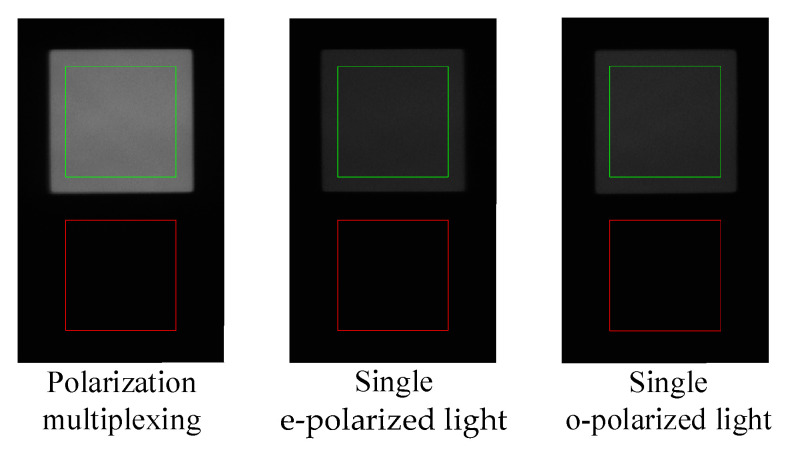
The captured images of the square target at 632.8 nm.

Then, the actual throughput of the proposed polarization multiplexing design and the traditional single polarization light design are compared in this paper. The evaluation factor used is the average Digital Number (DN) index of the detector within the effective area (green box area of the images), while the image signal-to-noise ratio (SNR) is evaluated using the same size of the red box area of the image. In the tests, len1 and lens2 temporarily use a single lens here, which can be optically optimized in the future. Additionally, further details of the system parameters can be found in Table 1. In the traditional design, cross polarizers are used before and after the AOTF device as shown in Figure 10, and the baffle used is wider to eliminate interference from the other polarization light. The polarizer used in the system has a transmittance of approximately 86% (GCL-050003). The imager uses a camera (Basler acA640-120gm) with 50 mm focal length lens (PENTAX B5014A). The light source is also the wide-spectrum light source composed of an integrating sphere and a collimator. The target is a hollow square target. Throughout the experiment, the camera maintained a consistent exposure time of 20 ms (milliseconds) and a fixed gain of 300. The captured images are listed in Figure 11, which show that the proposed polarization multiplexing design can effectively improve the system throughput by over 2.6-fold, as indicated in Table 3. By eliminating the losses incurred by the cross polarizers, the resulting increase in throughput is nearly twice as much as predicted by the theoretical calculations in Section 2.3. Simultaneously, the efficiency of conventional designs in separately collecting o light and e light is comparable.

This paper also includes a comparison of the SNR of images captured by various systems. The SNR can be computed using the following formula:

(19) R=20lgDNsDNn

where $DN_{s}$ and $DN_{n}$ are the DN values of the signal and noise, respectively. The variable R represents the magnitude of the image SNR. The results demonstrate that the proposed polarization multiplexing design improves the image SNR by approximately 8 dB, as illustrated in Table 3.

### 3.3. Test and Analysis of Spatial Resolution

**Figure 12:**
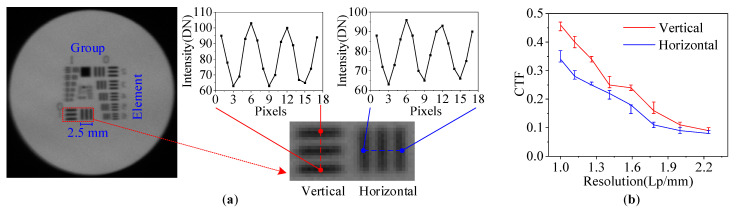
Resolution test and analysis results. (**a**) Captured image of USAF-1951 resolution target with polarization multiplexing application. (**b**) CTF analysis results.

In addition, spatial resolution represents a crucial characteristic of the imaging system. To assess the image quality in the polarization multiplexing system, we conducted a comprehensive evaluation employing the USAF-1951 resolution target as the reference. Figure 12a displayed the captured image, revealing a notable improvement in quality compared to previously published works [18,35]. For quantitative assessment, we utilized the contrast transfer function (CTF) defined as follows:

(20) $$F=\frac{DN_{\max}-DN_{\min}}{DN_{\max}+DN_{\min}}$$

where $DN_{\max}$ and $DN_{\min}$ are the maximum and minimum DN values along the orthogonal line direction as shown in Figure 12a, respectively. The variable F denotes the CTF value. Furthermore, we obtained the CTF analysis results in both horizontal and vertical directions, displayed in Figure 12b. The horizontal direction corresponds to the AOTF diffraction direction. The spatial resolution of the USAF-1951 target can be determined as follows:

(21) $$\mathrm{Resolution}=2^{Group+\frac{\left(Element-1\right)}{6}}$$

where Group is the group number and Element is the element number as Figure 12a. In the red dot wireframe (Group=0, Element=1), the corresponding single linewidth is 0.5 mm, indicating a resolution of 1 Lp/mm. The CTF analysis results show that the horizontal resolution is slightly higher than the vertical resolution as shown in Figure 12b. This discrepancy is primarily influenced by polarization multiplexing, resulting from the distinction between o and e light [36,37]. In future research, we aim to enhance this aspect by optimizing the geometric parameters of the AOTF device (as detailed in Section 2.2) and optimizing the lens group of the overall optical system.

## 4. Conclusions

In summary, we propose a novel design to address the low throughput of traditional AOTF spectral imaging systems. Our design utilizes polarization multiplexing to simultaneously capture ±1 order light of the AOTF device, resulting in a more than two-fold increase in system throughput. The experimental results validate the effectiveness of our design in improving system throughput and enhancing imaging SNR by approximately 8 dB. Moreover, our proposed design offers simplicity in structure compared to previously published works. Additionally, it is optimized for concerns such as inconsistent spectral response, real aperture angle compression, and transmission stray light interference. This research offers a valuable contribution to the field of AOTF spectral imaging systems in target detection applications. However, it should be noted that the polarization multiplexing design may lead to a reduction in spatial resolution, especially in the AOTF diffraction direction, which necessitates further efforts to overcome in future studies.

In addition, AOTF devices used in polarization multiplexing applications require a special design that does not follow the parallel tangent principle. This paper introduces an optimization strategy for arbitrary AOTF devices, which can achieve similar spectral effects without incurring high costs or long manufacturing periods. The test results demonstrate that the optimization strategy effectively corrects the spectral inconsistency of ±1 order light from the AOTF device at the same tuning frequency. This work is not only applicable to the proposed polarization multiplexing system, but also beneficial to AOTF polarization spectroscopy detection systems [38] and other AOTF detection systems that collect ±1 order light simultaneously.

## Figures and Tables

**Table 1:** Example system parameters of the proposed design.

Parameters	The 1st AOTF	The 2nd AOTF (Optimization Strategy)
$2\omega_{0}\;({}^{\circ})$	±7.5	±7.5
$D_{\mathrm{input}}\;\left(\mathrm{mm}\right)$	5.2	5.2
$f_{1}\;(\mathrm{mm})$	75	75
$L_{2}^{1}\;(\mathrm{mm})$	66.8	67.1
$D_{\mathrm{AOTF}}\;\left(\mathrm{mm}\right)$	20	20
$\theta_{\mathrm{AOTF}}\;({}^{\circ})$	4.0	4.0
$f_{2}\;\left(\mathrm{mm}\right)$	75	75
$D_{\mathrm{block}}\;\left(\mathrm{mm}\right)$	5.2	5.3
$D_{\mathrm{imager}}\;\left(\mathrm{mm}\right)$	15.9	16.1

**Table 2:** Theoretical throughput data and comparison results.

AOTF Device	The 1st AOTF			The 2nd AOTF		
Detection Mode	Polarization Multiplexing	Single e Light	Single o Light	Polarization Multiplexing	Single e Light	Single o Light
Theoretical throughput within 620–650 nm	78,090	38,458	39,119	77,680	37,979	38,402
Maximum diffraction efficiency	33.3%	32.4%	32.6%	33.1%	31.8%	32.4%
Ratio of throughput improvement		2.03	2.00		2.05	2.02

**Table 3:** The response DN values of the captured images.

Parameter	Test 1	Test 2	Test 3	Average
$\mathrm{Signal}\;\mathrm{of}\;\mathrm{polarization}\;\mathrm{multiplexing}\;(DN_{s}^{pm}$)	109.7	109.5	109.5	109.5
$\mathrm{Signal}\;\mathrm{of}\;\sin\mathrm{gle}\;e-\mathrm{polarized}\;\mathrm{light}\;(DN_{s}^{e}$)	38.1	38.6	38.3	38.3
$\mathrm{Signal}\;\mathrm{of}\;\sin\mathrm{gle}\;o-\mathrm{polarized}\;\mathrm{light}\;(DN_{s}^{o}$)	41.5	42.0	41.9	41.8
$DN_{s}^{pm}/DN_{s}^{e}$	2.88	2.84	2.86	2.85
(Eliminating loss of polarizers)	(2.13)	(2.10)	(2.11)	(2.11)
$DN_{s}^{pm}/DN_{s}^{o}$	2.64	2.61	2.61	2.62
(Eliminating loss of polarizers)	(1.96)	(1.93)	(1.93)	(1.94)
$\mathrm{Noise}\;\mathrm{of}\;\mathrm{polarization}\;\mathrm{multiplexing}\;(DN_{n}^{pm}$)	4.9	4.7	4.7	4.8
$\mathrm{Noise}\;\mathrm{of}\;\sin\mathrm{gle}\;e-\mathrm{polarized}\;\mathrm{light}\;(DN_{n}^{e}$)	4.2	4.6	4.4	4.4
$\mathrm{Noise}\;\mathrm{of}\;\sin\mathrm{gle}\;o-\mathrm{polarized}\;\mathrm{light}\;(DN_{n}^{o}$)	4.4	4.9	4.8	4.7
$R^{pm}$/dB	27.0	27.4	27.3	27.2
$R^{e}$/dB	19.1	18.5	18.8	18.8
$R^{o}$/dB	19.5	18.7	18.8	19.0

## Data Availability

Not applicable.
